# Environmental factors and antimicrobial efficacy: the impact of temperature and humidity on material surfaces

**DOI:** 10.1128/spectrum.00960-25

**Published:** 2025-10-16

**Authors:** Han Cheng, Jie Chen, Hao Yu, Bin Sun, Jialiang Zhou, Guoyi Wu

**Affiliations:** 1 Shanghai Public Health Clinical Center, Fudan University 12478, Shanghai, China; 2 State Key Laboratory of Advanced Fiber Materials, College of Material Science and Engineering, Donghua University 12475, Shanghai, China; 3 Jiangsu Gem Advanced Fiber Materials Research Institute Co. Ltd., Nantong, China; Fujian Agriculture and Forestry University, Fuzhou City, Fujian, China

**Keywords:** antimicrobial efficacy, ISO testing standards, environmental variability, pathogen survival kinetics, copper composites

## Abstract

Current International Standards Organization (ISO) for testing antimicrobial materials often lack regulatory approval for clinical use because they do not accurately reflect real-world environmental conditions. In this study, we systematically evaluated the effects of temperature (4°C–37°C) and humidity (15%–100% relative humidity) on microbial survival (*Staphylococcus aureus*, *Escherichia coli*, HCoV-OC43, and influenza virus) and the efficacy of various antimicrobial surfaces (stainless steel, copper, polyethylene terephthalate [PET], and Cu₂O@ZrP-modified PET) using established ISO standards, including ISO 21702, 18184, 22196, and 20743. We found that pathogen survival declined sharply with increasing temperature, while the effects of humidity varied by material. Copper and Cu₂O@ZrP-PET surfaces achieved greater than 99% viral and bacterial inactivation within 60 min; however, their activity was delayed by two to threefold at 4°C. The effects of humidity were material-dependent. Non-porous copper maintained consistent efficacy across humidity levels, whereas PET/Cu₂O@ZrP exhibited enhanced antibacterial activity under low humidity. Mechanistically, reactive oxygen species generation correlated with efficacy changes across conditions. We advocate revising ISO protocols to include dynamic environmental parameters and propose a tiered classification of antimicrobial efficiency that aligns with clinical demands. This framework addresses the discrepancy between laboratory tests and real-world performance, enabling the robust evaluation of antimicrobial materials for clinical and public health applications.

**IMPORTANCE:**

This study addresses a critical gap in our understanding of how real-world environmental conditions affect the performance of antimicrobial materials. Current International Standards Organization (ISO) testing standards fail to adequately account for temperature and humidity variations, leading to discrepancies between laboratory results and real-world effectiveness. Our findings demonstrate that both temperature and humidity significantly impact pathogen survival and antimicrobial efficacy, with important implications for material selection in healthcare, public spaces, and pandemic preparedness. By systematically evaluating these environmental factors across different material types, we provide evidence-based recommendations for revising international testing protocols. This work is essential for ensuring that antimicrobial materials perform as expected when deployed in actual environments, potentially saving lives by improving the reliability of these critical defense mechanisms against infectious diseases.

## INTRODUCTION

Infectious disease pandemics and epidemics are significant and ongoing global threats. Over the last century, the risk of emerging infectious diseases has steadily increased alongside the rise in international travel, cross-border trade, and livestock farming. Additionally, epidemic and pandemic waves are linked to the growing human population density and evolving interactions between humans and wild animals (1). The coronavirus disease 2019 (COVID-19) pandemic, caused by severe acute respiratory syndrome coronavirus 2, has resulted in substantial morbidity and mortality, as well as serious social and economic disruptions worldwide. The COVID-19 pandemic has emphasized the importance of stopping the spread of pathogens (2). Infectious diseases are caused by microorganisms, such as bacteria, viruses, fungi, or parasites. Therefore, the development of advanced antimicrobial materials and coatings capable of preventing viral and bacterial transmission is essential to protect humans from emerging infectious microorganisms. In order to prevent the spread of microorganisms during the past decades, substantial scientific research and developments related to antimicrobial materials and surfaces have increased continuously (3).

Antimicrobial materials are of great significance, but the test results for judging the antibacterial and antiviral functions of materials often vary significantly from the actual application effects. This has resulted in regulatory authorities in different countries rarely approving these materials that claim to have antimicrobial functions as medical devices for clinical use. In order to analyze the shortcomings of the existing antibacterial and antiviral testing methodologies for materials, we need to find out the causes and mechanisms leading to this problem and provide theoretical and data support for improving testing methods to meet the requirements for antimicrobial material approval.

There are four commonly used International Standards Organization (ISO) testing methodologies, including ISO 21702:2019 for testing the antiviral properties of non-porous material surfaces (4), ISO 18184:2019 for evaluating the antiviral efficacy of textiles (5), ISO 22196:2011 for assessing the antibacterial efficacy of non-porous surfaces (6), and ISO 20743:2013 for testing the antibacterial performance of porous textile surfaces (7). Although other testing methods are also employed, these testing methods take into account the surface characteristics of the materials, the representativeness of the inoculated pathogenic microorganisms, and the feasibility of the testing process. However, the results from these tests often differ significantly from actual usage outcomes. This discrepancy may arise partly because standard methods frequently overlook real-world environmental factors, such as temperature, humidity, contaminant levels, and light exposure. For a better understanding of the differences between these standard testing methods, some details of these standard methods are shown in Table 1.

**TABLE 1:** Comparison between different ISO antibacterial and antiviral testing methods^*a*^

Testing standards	ISO 22196	ISO 20743	ISO 21702	ISO 18184
Samples	Plastics/non-porous	Porous/textiles	Non-porous/plastics	Porous/textiles
Microbiological samples	*S. aureus/E. coli*	*S. aureus/K. pneumoniae*	Influenza A virus/Feline calicivirus	Influenza A virus/Feline calicivirus
Inoculation/detection method	Bacterial suspension applied to surface, covered with sterile PE film; recovery by neutralizing solution, dilution, plating	Absorption, transfer, or printing methods; recovery by shaking/sonication, dilution, plating	Viral suspension (1–5 × 10^7^ PFU/mL in PBS) applied (0.4 mL), covered with PE film; recovery by SCDLP broth, titration by host-cell assay (TCID_50_ or PFU)	Viral inoculum (1–5 × 10^7^ PFU/mL in PBS) applied to specimen; recovery by elution, titration, host-cell assay (TCID_50_ or PFU)
Incubation time (h)	24 ± 1	18–24	24	2–24
Temperature (°C)	35 ± 1	37 ± 2/20 ± 2	25 ± 1	25
Relative humidity	90%	N/A (absorption method)/N/A (transfer method)/70% (printing method)	90%	N/A
Film cover	Yes	No	Yes	No

^*a*^ “N/A” indicates not available.

Among these environmental factors, temperature is a well-established factor influencing microbial survival on non-antimicrobial surfaces, with elevated temperatures accelerating pathogen inactivation (8–10). Similarly, humidity modulates pathogen persistence, though its effects vary across material types (11, 12). However, the interplay between environmental variables (temperature, humidity) and antimicrobial material efficacy remains understudied. Current ISO testing protocols often neglect these parameters, leading to potential discrepancies between laboratory results and real-world performance (13). This study systematically evaluates temperature (4°C–37°C) and humidity (15%–100% relative humidity [RH]) effects on microbial survival and antimicrobial material efficacy across porous (polyethylene terephthalate [PET]) and non-porous (copper) surfaces. Furthermore, we propose mechanistic insights by correlating environmental conditions with reactive oxygen species (ROS) generation—a key bactericidal pathway—to establish a framework for revising ISO standards.

## MATERIALS AND METHODS

### Bacterial strains and growth media

*Staphylococcus aureus* (*S. aureus*, ATCC 25923) and *Escherichia coli* (*E. coli*, ATCC 25922) were procured from the Shanghai Public Health Clinical Center. Strains were subcultured in tryptic soy broth (TSB) under aerobic conditions at 37°C for 24 h to reach the logarithmic growth phase. Bacterial suspensions were prepared in phosphate-buffered saline (PBS, pH 7.4) containing 0.1% (vol/vol) Tween-80, with an initial concentration adjusted to 1 × 10^9^ colony-forming units per milliliter (CFU/mL) using optical density calibration (OD600). Subsequent dilutions to target concentrations (e.g., 1 × 10^6^ CFU/mL) were performed following McFarland’s standard turbidity method, validated via serial plating on TSB agar.

### Cells and viruses

Human coronavirus OC43 (HCoV-OC43, KBPV-VR-8) and Influenza virus (A/PR/8/34 H1N1) were obtained from the Shanghai Public Health Clinical Center. Baby hamster kidney cells (BHK-21) were used to propagate HCoV-OC43, while the influenza virus was propagated in Madin-Darby canine kidney (MDCK) cells. Host cells were cultivated in MEM medium containing 10% fetal bovine serum, 1% 10 mM HEPES in 0.85% NaCl, and 100 U/mL penicillin–100 µg/mL streptomycin. These cells were maintained at 37°C with 5% CO_2_. For viral propagation, BHK-21 cells were infected with HCoV-OC43, and MDCK cells were infected with PR8, both incubated at 37°C in the presence of 5% CO_2_. Following the appearance of cytopathic effects, the cells were subjected to a freeze/thaw cycle. Cell debris was removed via centrifugation, and the supernatant was collected and stored at −80°C until use and titrated by plaque assay following standard procedures.

### Test materials surface preparation

Four test materials were chosen to simulate surfaces typically found within the ISO standards. The two non-porous surfaces were stainless steel (24-gauge 304 stainless steel) and copper foil (99.9% pure alloy C11000), both of which were purchased from the Shanghai Metal Company of China. The two porous surfaces, 100% polyester–PET fibers and PET/Cu_2_O@ZrP fibers, were prepared by Dr. Jialiang Zhou from Donghua University (14). The PET fibers containing Cu_2_O@ZrP prepared by *in situ* polymerization and melt spinning processes exhibit broad-spectrum antibacterial ability (15). Two non-porous materials were cut into 4.7 cm × 4.7 cm coupons that were washed and autoclaved at 121°C for 15 min allowed to dry prior to each experiment. For porous samples, aliquots of 500 mg each were prepared but not subjected to sterilization with steam at 121°C for 15 min prior to use, to avoid the impact of high temperature on the fiber structure. In addition, these materials are highly relevant to public health settings. Stainless steel and copper are widely used in hospitals for their hygiene and durability, while PET is common in both healthcare and everyday environments. Modified PET materials, such as Cu₂O@ZrP-PET, represent next-generation solutions designed to enhance contamination control in areas with high risks of microbial transmission.

### Testing of antimicrobial effect of the surfaces

To evaluate the activity of these antimicrobial materials, appropriate standard test methods are necessary. Currently, the ISO 22196, 21702, 20743, and 18184 test standards are used by most manufacturers to assess antibacterial and antiviral activity on plastics and other non-porous surfaces, as well as textiles. These protocols share a similarity, where surfaces are tested using a liquid bacterial or virus inoculum, which is cultivated at 37°C for 2 to 24 h. Viable bacteria or virus counts are evaluated and compared to counts on untreated surfaces (16). Antibacterial and antiviral assays were carried out in accordance with the applicable ISO standard methods, selected based on whether the test material was porous or non-porous. Experimental parameters, including ambient temperature, humidity, and inoculation period, were adjusted as required. A comparison of the four standardized testing methods is provided in Table 1. The following sections describe the detailed procedures for each ISO standard, including the test materials, target microorganisms, inoculation methods, incubation conditions, and assessment of antibacterial or antiviral activity.

### ISO 22196:2011 measurement of antibacterial activity on plastics and other non-porous surfaces

Performance testing of the non-porous material surfaces was principally conducted in accordance with ISO 22196:2011. ISO 22196:2011 specifies a method for evaluating the antibacterial activity of plastics and other non-porous surfaces. In this procedure, a test bacterial suspension, typically *E. coli* and *S. aureus*, is prepared at a defined concentration of 10^5^–10^6^ CFU/mL. A 0.4 mL volume of the inoculum is placed onto the surface of the test specimen, which is then covered with a sterile film to ensure uniform contact and to prevent evaporation. Control specimens without antibacterial treatment are prepared in parallel. The incubation is then done under defined temperature conditions at 35°C ± 1°C and a RH of not less than 90%, for a defined contact period (commonly 24 h). After incubation, bacteria are recovered by washing the specimens with a neutralizing solution. The resulting bacterial suspension is serially diluted and plated to determine the number of viable colonies. The antibacterial activity (R value) is calculated by comparing the logarithmic reduction in viable bacterial counts between the test specimen and the control. An R value greater than 2 is generally considered to indicate significant antibacterial activity. It represents a simple and inexpensive protocol by applying the bacterial suspension with a known volume and concentration covered under a polyethylene film on the surfaces.

### ISO 20743:2021 textiles—determination of antibacterial activity of textile products

ISO 20743:2021 specifies test methods for determining the antibacterial activity of antibacterial finished textiles. The procedure involves inoculating textile specimens with a defined bacterial suspension, commonly *S. aureus* (Gram+) and *Klebsiella pneumoniae* (Gram−). Several test options are provided, including the absorption method, the transfer method, and the printing method, which differ in how bacteria are introduced to the textile surface. The most used ISO 20743 test procedure is the absorption method in which the test bacterial suspension is inoculated directly onto specimens. The test bacterial suspension is normally at a bacterial concentration of 10^5^–10^6^ CFU/mL and 0.2–0.5 mL of suspensions are incubated under controlled environmental conditions, usually at 37°C and high humidity, for a specified period (typically 18–24 h). After incubation, viable bacteria are recovered from the textile specimens by shaking or sonication in a neutralizing solution. The bacterial suspension is then serially diluted, plated, and cultured to determine colony counts. Antibacterial activity is expressed as a reduction value (log reduction), comparing the bacterial count from the treated specimen with that from the untreated control. Higher reduction values indicate stronger antibacterial effects. Although the printing method of ISO 20743 standard defined the testing conditions at a relative humidity (RH > 90%), for the absorption or transfer method of ISO 20743, the relative humidity during inoculation was not prescribed (17, 18).

### ISO 18184:2019 textiles—determination of antiviral activity of textile products

ISO 18184:2019 specifies a method for determining the antiviral activity of textile products. In this procedure, test specimens are inoculated with a defined viral suspension, commonly including influenza viruses, feline calicivirus, or other relevant strains. Untreated textile specimens serve as negative controls. A measured volume of the viral inoculum is applied directly to the surface of the textile specimen. To ensure consistent contact, the inoculum is covered and maintained under controlled environmental conditions, typically at room temperature, for a specified contact period (usually 2 to 24 h). Whereas the environmental humidity during incubation was not defined in the ISO 18184. Following incubation, the virus is recovered from the textile specimen by elution into a suitable neutralizing medium. The eluate is then titrated and assayed using permissive host cells to determine the residual infectious viral particles, generally quantified as tissue culture infectious dose (TCID_50_) or plaque-forming units (PFU) (19). The remaining infectious virus was counted, and the reduction rate was calculated by the comparison between the antiviral product test specimen and the control specimen by common logarithm. A higher reduction indicates stronger antiviral activity.

### ISO 21702:2019 measurement of antiviral activity on plastics and other non-porous surfaces

ISO 21702:2019 specifies a method for measuring the antiviral activity of plastics and other non-porous surfaces. The test specimens are inoculated with a defined viral suspension, such as influenza virus, feline calicivirus, or other relevant strains, while untreated specimens are used as controls. Normally, viral inoculum was collected and suspended in PBS at 1–5 × 10^7^ plaque-forming units per milliliter. An aliquot (0.4 mL) of the virus suspension is placed onto the specimen surface and covered with a disinfected PE film (4 × 4 cm) to ensure uniform distribution and to prevent evaporation. The inoculated specimens are then incubated under controlled environmental conditions, typically at 25°C and high relative humidity of 90% or higher, for a specified contact time (commonly 24 h). After incubation, the virus was washed out of the specimen with 10 mL of SCDLP broth, and the live virus was subsequently titrated and assayed using a suitable host cell system to determine the number of infectious viral particles. This standardized method enables reproducible and comparable evaluation of antiviral performance in plastics and other non-porous materials, supporting both research and product validation.

### Preparation of controlled RH chamber

To create controlled RH chambers, various salt solutions were prepared as follows: 230 g of lithium chloride (LiCl) was dissolved in 100 mL of heated water to create a saturated LiCl solution. This solution generates a relative humidity of 15%. A 230 g of potassium carbonate (K₂CO₃) was dissolved in 100 mL of heated water to create a saturated K₂CO₃ solution, which stabilizes at 50% RH. A total of 250 g of potassium sulfate (K₂SO₄) was dissolved in 100 mL of water to create a saturated K₂SO₄ solution, resulting in 100% RH. The prepared salt solutions were placed at the bottom of glass desiccators. The desiccators were sealed with Vaseline around the rim to ensure a tight, waterproof seal. They were then left undisturbed for 24 h to allow the RH to stabilize. Data loggers were used to monitor the RH and temperature inside the desiccators, with a precision of ±1°C for temperature and ±2% for RH, ensuring accurate monitoring throughout the experiment.

### Fluorescent reactive oxygen species detection

The production of ROS by PET/Cu_2_O@ZrP fibers was examined using 2′,7′-dichlorofluorescin diacetate (DCFH-DA) dye (Sigma Aldrich). The dye was added to bacterial suspensions and incubated for 30 min in the dark. Prior to testing, material samples were sterilized using ultraviolet light. They were then inoculated with an *E. coli* suspension at a concentration of 10^8^ CFU/mL in PBS and co-incubated for 6 h. The cell suspension was washed and centrifuged at 4,000 rpm for 10 min, after which the supernatant was discarded. The cells were washed twice with PBS. Subsequently, 1 mL of DCFH-DA (20 µM/L in 0.9% NaCl) was added and incubated for 30 min at 37°C in the dark, followed by two washes with PBS. The cells were then resuspended in 0.9% NaCl. Fluorescent measurements were obtained by exciting at 502 nm and collecting emission signals at 530 nm.

### Statistical analysis

The data are presented as mean ± standard deviation, derived from multiple independent experiments. Statistical analyses and graphical representations were performed using GraphPad Prism 9 (GraphPad Software, San Diego, USA). Statistical significance was determined as indicated in the figure legends, with significance set at a *P* value of <0.05.

## RESULTS

### Viruses and bacteria can survive longer at low temperatures on material surfaces

To study the effect of temperature on the survival of viruses and bacteria on porous and non-porous surfaces, the above four ISO standard testing methodologies were used with varying inoculation temperature conditions. Stainless steel was chosen as the representative non-porous material, and polyester (PET) as the representative porous material. The coronavirus OC43 strain and H1N1 influenza virus PR8 strain were used as representative viruses, while *E. coli* and *S. aureus* were used as representative bacteria. The study was conducted over a 72 h span at ambient humidity (25% relative humidity) for viruses, while for bacteria, the inoculation span was 168 h. Different viruses or bacteria were inoculated onto material surfaces at 4°C, 25°C, or 37°C, and the viability of each microbe was measured at different time intervals.

**Fig 1:**
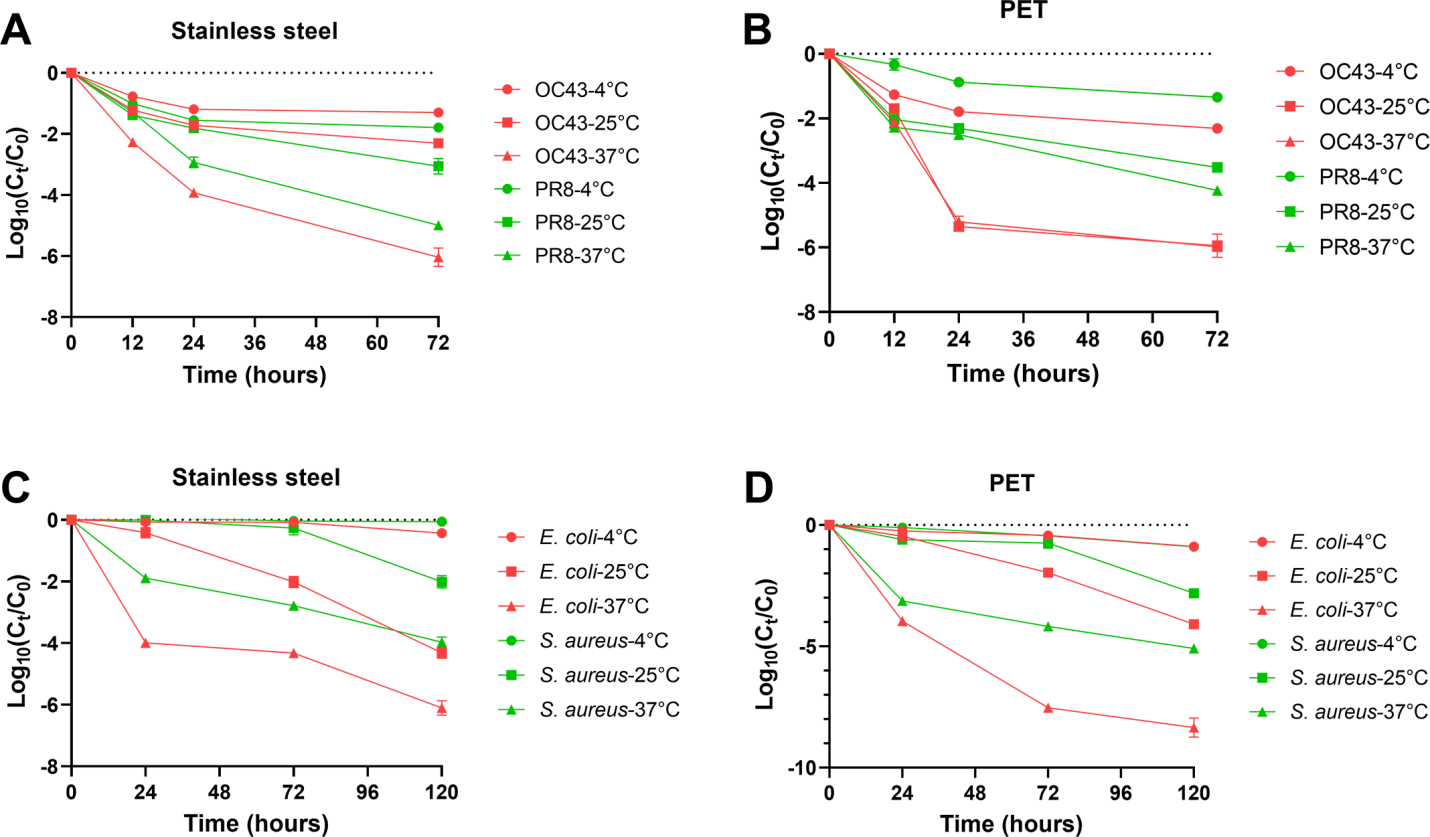
Survival of different viruses and bacterial species on porous or non-porous surfaces under different ambient temperatures. (**A and B**) Different viruses on stainless steel and PET over 72 h, and (**C and D**) different bacterial species on stainless steel and PET over 120 h.

Approximately 6.33 Log_10_ TCID_50_/mL of OC43 or 6.03 Log_10_ PFU/mL of PR8 viruses were inoculated onto the non-porous surfaces, and then the viral viability was measured at different time intervals at 4°C, 25°C, or 37°C, respectively, as described previously (20, 21). Similar viral viability dynamics of OC43 and PR8 were observed on stainless steel surfaces at each temperature setting post-inoculation (Fig. 1A). Specifically, both OC43 and PR8 viruses were relatively stable at 4°C for up to 72 h compared to higher temperatures, with a slight viral titer decrease in the first 6 h, subsequently reaching a new steady-state level at a titer of 5.28 Log_10_ TCID_50_/mL and 4.20 Log_10_ PFU/mL, respectively. Notably, the highest temperatures at 37°C significantly accelerated the decay of OC43 and PR8 viruses, with a 4.03 log and 2.40 log decrease in viable virus counts, respectively, in the first 24 h. This led to the loss of more than 99.99% viable OC43 or PR8 viruses after 72 h at 37°C.

Compared with non-porous surfaces, the decay rates of viruses on PET material surfaces are similar at 25°C and 37°C (Fig. 1B). The viable OC43 and PR8 virus counts decreased more than 99% after 24 h of inoculation at 25°C and 37°C and continued to drop to more than 99.99% or 99.9% death after 72 h, respectively. However, the drop in mortality rates for both OC43 and PR8 on a PET surface at 4°C is 98.3% and 86.7% within 24 h, respectively, which means that a large number of viruses survive. Even after 72 h, both viruses still had a survival rate of more than 1/1,000, which indicates the stable survival of viruses on PET at low temperature.

Survival of *E. coli* and *S. aureus* on non-porous and porous surfaces was tested according to ISO 22196 and ISO 20743, respectively. The testing conditions were set according to ISO standards, and the inoculation temperatures were adjusted to 4°C, 25°C, and 37°C to study the effect of temperature on the survival of bacteria on the material surface. Similar to viruses, the survival rate of bacteria on the surfaces of both materials is significantly affected by the inoculation temperature. It shows that the survival rate of two bacterial strains at 37°C is significantly lower than at 25°C after 24 h, whether on stainless steel surfaces or PET surfaces (Fig. 1C and D). However, under low temperature conditions at 4°C, the survival rates of different bacteria didn’t decrease significantly even after 120 h of inoculation on the material’s surfaces. The survival rate on stainless steel or PET is different between the two bacterial strains at 72 and 120 h at 25°C, suggesting that temperature changes have different effects on the survival rates of different bacteria. These results indicated that bacteria are stable on stainless steel or PET surfaces at 4°C, whereas bacteria’s survival rates decreased rapidly under higher temperatures at 37°C.

Taken together, the survival of viruses and bacteria on non-porous or porous material surfaces is affected by the ambient temperature. Generally, the higher the temperature, the lower the survival rate. This is consistent with findings in previous studies (22, 23).

### The ambient humidity has an influence on the survival of microbes on porous and non-porous surfaces

The survival of bacteria and viruses on material surfaces is significantly influenced by ambient humidity. To evaluate whether current testing methods can accurately capture the effects of varying humidity levels on microbial survival, we assessed the viability of these pathogens on material surfaces under different humidity conditions at 25°C. The experiments were conducted following the standardized methodologies described in ISO 20743, ISO 18184, ISO 21702, and ISO 22196.

**Fig 2:**
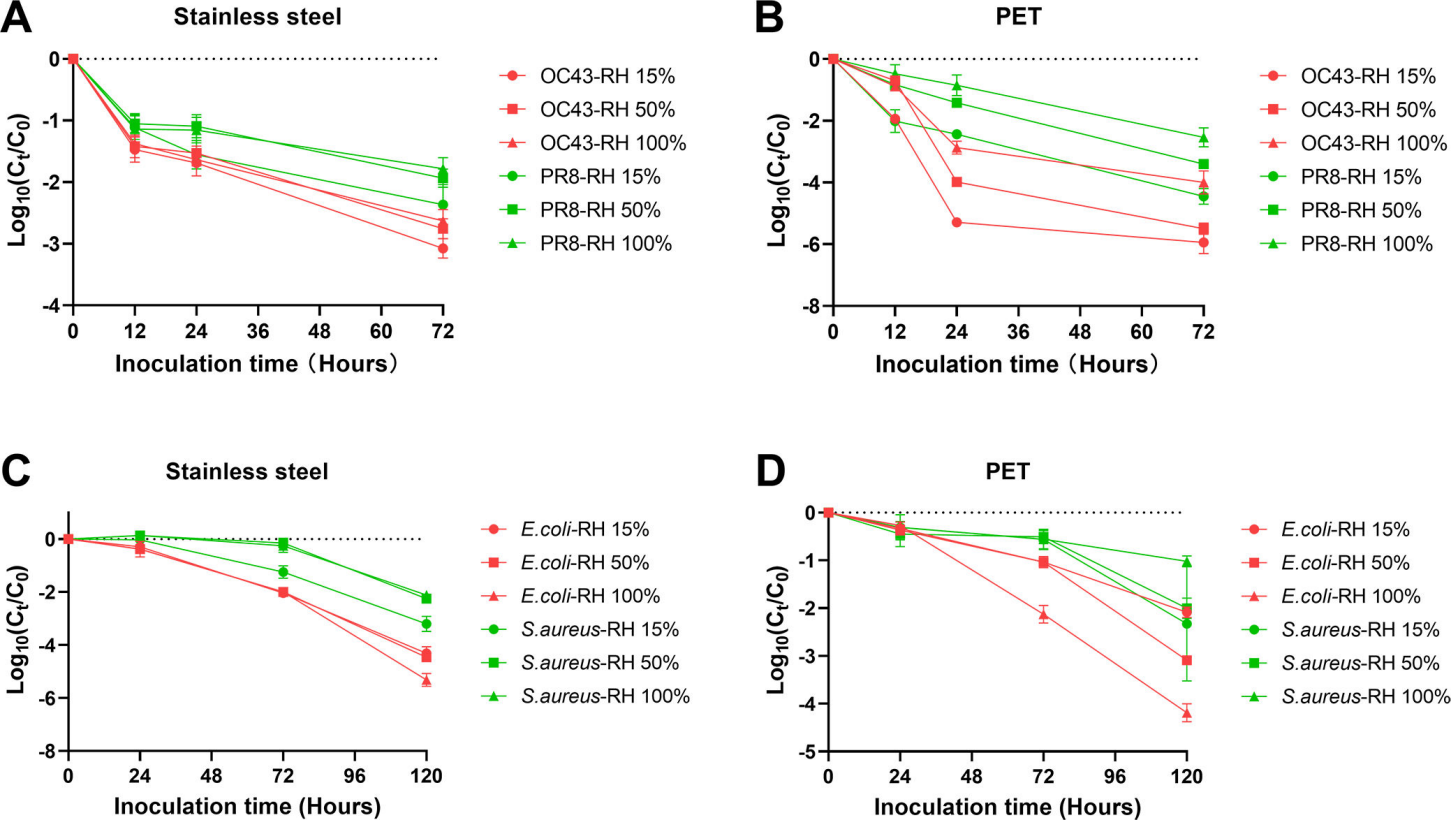
Survival of different viruses and bacterial species on porous or non-porous surfaces under different RH. (**A and B**) Different viruses on stainless steel and PET over 72 h, and (**C and D**) different bacterial species on stainless steel and PET over 120 h.

We assessed the survival rates of PR8 and OC43 on stainless steel over 72 h using the ISO 20743 method under three humidity conditions. PR8 consistently exhibited significantly higher survival rates than OC43. However, there was no statistically significant difference in survival rates across humidity levels for either virus (Fig. 2A). In contrast to the ISO 20743 method for testing virus survival on non-porous surfaces, the ISO 18184 method, used for evaluating virus survival on PET non-woven fabric surfaces, revealed notable differences in the survival of OC43 and PR8 under varying environmental humidity conditions. Specifically, within the first 24 h on the PET surface, lower humidity was associated with a significantly reduced survival rate for both virus strains. Furthermore, after 72 h of inoculation, the survival rates of viruses under 15% RH were significantly lower than those under 100% RH (Fig. 2B).

Similarly, when using the ISO 21702 method to assess the 120 h survival of bacteria on stainless steel surfaces under varying humidity conditions, it was found that neither *E. coli* nor *S. aureus* survival was significantly affected by changes in ambient humidity (Fig. 2C). In contrast, bacterial survival testing on PET surfaces using the ISO 22196 method revealed that lower humidity levels were associated with reduced survival rates for both *S. aureus,* but *E. coli* can live longer on PET under lower humidity (Fig. 2D), which is consistent with the results reported by previous studies (24).

These findings indicate that under different environmental humidity conditions, the choice of testing methodology can influence the observed survival rates of viruses and bacteria on material surfaces. Moreover, the impact of humidity on microbial survival appears to be more pronounced on porous materials compared to non-porous surfaces. Similar phenomena of humidity affecting survival have also been found in bacterial (25) or virus (26, 27) survival tests on other material surfaces, but the impact of humidity varies in different test durations and methods. Especially, environmental humidity was also found to influence the antimicrobial testing results of materials (28).

### Copper foil and PET/Cu_2_O@ZrP materials exhibit effective antibacterial and antiviral properties at ambient temperature and humidity

To investigate whether the testing results for the efficacy of non-porous and porous antimicrobial materials are influenced by changes in ambient temperature and humidity within the selected methodology procedures, we employed the four ISO standard methods mentioned above. These methods were first used to evaluate the antimicrobial effects of copper foil and PET/Cu_2_O@ZrP materials under controlled conditions: an inoculation time of 60 min, a temperature of 25°C, and a relative humidity of 50%. Both copper foil (29, 30) and PET/Cu_2_O@ZrP were demonstrated to effectively kill bacteria and viruses on their surfaces. Stainless steel and PET fiber were used as negative control materials for copper foil and PET/Cu_2_O@ZrP, respectively.

**Fig 3:**
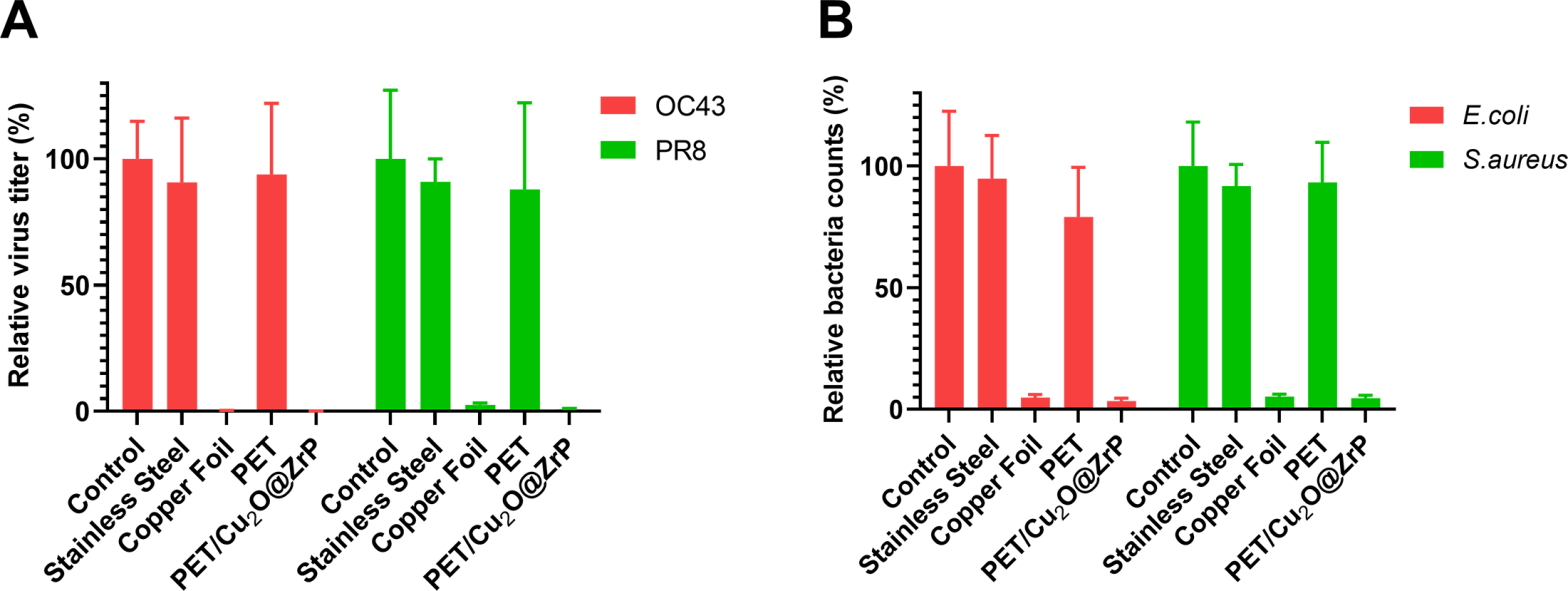
Antimicrobial efficacy of copper foil and PET/Cu_2_O@ZrP after 60 min of inoculation. (**A**) Copper foil and PET/Cu_2_O@ZrP can effectively kill OC43 and PR8 viruses in 60 min; (**B**) copper foil and PET/Cu_2_O@ZrP effectively kill *E. coli* and *S. aureus* bacterial strains in 60 min.

Approximately 60 min after inoculating OC43 or PR8, no significant differences were observed between the initial viable virus level (control) and viable virus levels on stainless steel or PET materials (Fig. 3A). However, in both the OC43 and PR8 groups, the virus levels on the copper foil and PET/Cu_2_O@ZrP surfaces were significantly lower compared to the control. Specifically, when compared to stainless steel, copper foil was able to destroy more than 99% of viable OC43 and 97% of PR8 within 60 min. Even more effectively, PET/Cu_2_O@ZrP demonstrated rapid virucidal activity, eliminating 99% of both OC43 and PR8 viruses within the same time frame, whereas PET showed no significant virucidal effect.

Similarly, we tested the viable bacterial levels on the copper foil and PET/Cu_2_O@ZrP surfaces and found that the survival rates of both bacterial strains were significantly reduced by approximately 95% after 1 h of incubation (Fig. 3B). In contrast, the survival of *E. coli* and *S. aureus* on stainless steel or PET showed no significant difference compared to the control group.

These results demonstrate that copper foil and PET/Cu_2_O@ZrP are fast-acting antimicrobial materials capable of rapidly killing bacteria and viruses within a short time frame.

### The antimicrobial efficacy of copper foil and PET/Cu_2_O@ZrP is faster and more effective under higher temperature conditions

In previous studies, researchers have reported varying speeds for the antimicrobial efficacy of copper, with some suggesting it can kill viruses or bacteria within 4 h, others within 2 h, and some even within minutes (31, 32). These discrepancies are likely due to differences in testing protocols, such as the type of microorganism inoculated, microbial load, inoculation sample volume, and temperature. Such variations make it challenging to draw definitive conclusions about the antimicrobial efficacy of specific materials. As a result, incubation temperatures in various standard testing methodologies are typically specified as 25°C or 37°C. However, these fixed temperatures do not adequately reflect the antimicrobial properties of materials under real-world ambient conditions. Therefore, we propose that evaluating the antimicrobial properties of materials requires testing under a range of temperature conditions. In this study, we investigated the antibacterial efficiency of materials at 50% relative humidity by setting the inoculation temperatures to 4°C, 25°C, and 37°C. We then assessed the materials’ antimicrobial efficacy at different time points after inoculation. Unlike other studies, we emphasize the clinical significance of rapid antimicrobial effects, particularly achieving a high killing rate within 30 min for viruses and 90 min for bacteria.

**Fig 4:**
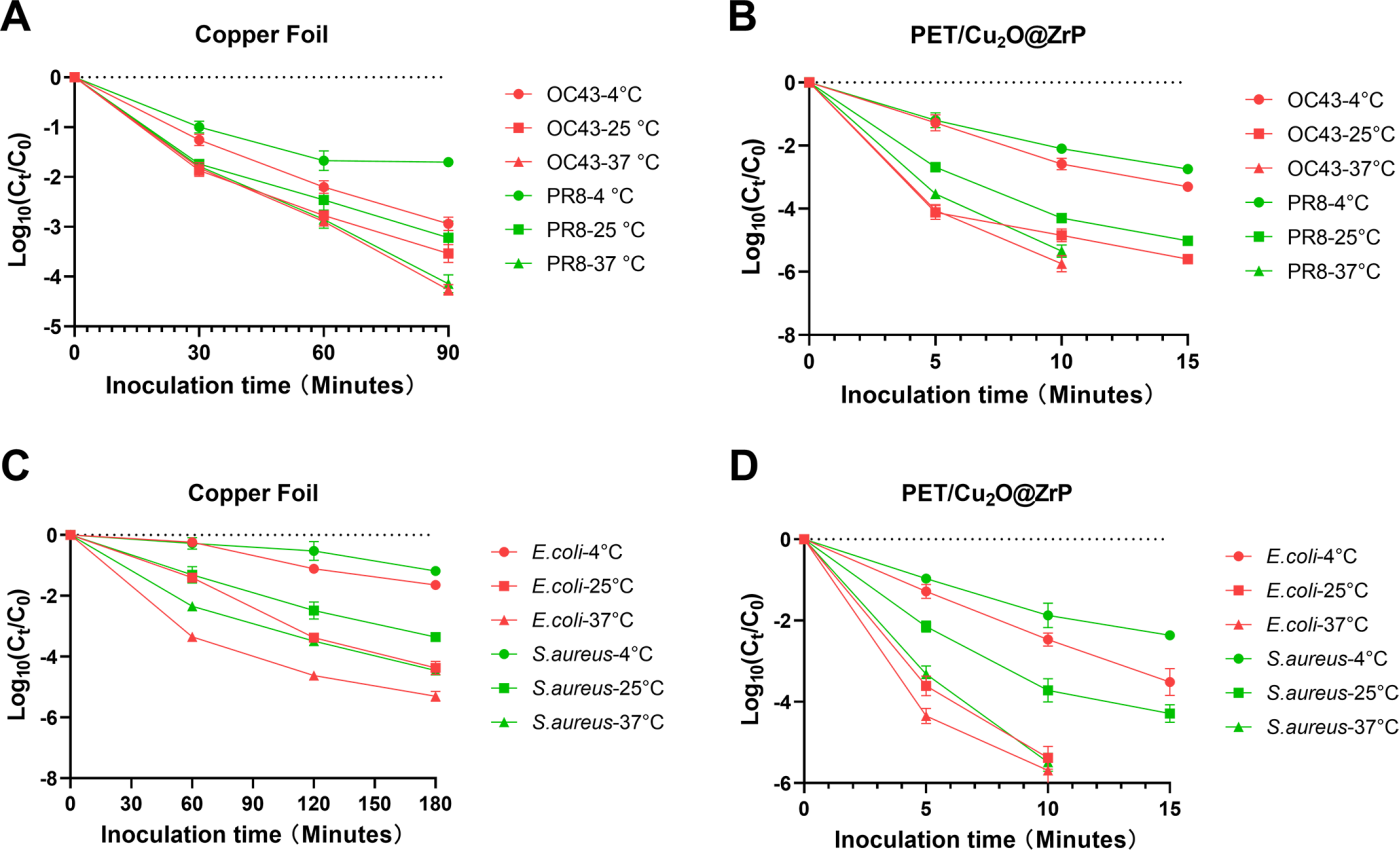
Environmental temperature has an influence on the antimicrobial testing results of material surfaces. (**A and B**) Viruses were killed faster under higher temperatures, either on non-porous or porous surfaces. (**C and D**) Different bacterial strains were killed faster under higher temperatures on antimicrobial material surfaces.

We tested pure copper foil using the ISO 22196 method to evaluate the effect of incubation temperature on the antiviral efficacy of non-porous antimicrobial materials. At 25°C, the copper foil inactivated over 99% of two virus strains within 30 min. However, at 4°C, both viruses showed greater tolerance, and the antiviral efficiency did not reach 99% against PR8 even after 90 min of exposure to the copper surface (Fig. 4A). The antiviral efficacy of PET/Cu_2_O@ZrP was significantly faster than that of copper foil, achieving a reduction of more than 99% of viruses at 25°C within just 5 min (Fig. 4B). The inoculation temperature was found to influence the antiviral performance of PET/Cu_2_O@ZrP, with its inhibitory effects diminished at 4°C for both virus strains and markedly enhanced at 37°C. These findings suggest that lower environmental temperatures reduce the antiviral efficacy of copper and its derivatives.

To determine whether temperature similarly affects the antibacterial properties of materials as their antiviral effects, *E. coli* and *S. aureus* were inoculated onto the surface of each material and incubated at 4°C, 25°C, and 37°C for the durations indicated in Fig. 4C and D, respectively. The results revealed that bacteria are more resistant to copper foil compared to viruses, as reducing the bacterial count by more than 99% required over 90 min. Similar to the temperature-dependent antiviral effects observed with the two materials, their antibacterial efficacy also decreased significantly under low-temperature conditions, respectively (Fig. 4C and D). Notably, *E. coli* was significantly inactivated faster than *S. aureus* on both the copper foil and PET/Cu_2_O@ZrP surfaces at each temperature.

### Low humidity conditions influence the antimicrobial effect of PET/Cu_2_O@ZrP but not copper foil, as tested by different ISO standard methods

Among the environmental factors considered in standard antimicrobial testing methods, humidity is also a critical parameter. Therefore, using the four ISO standard testing methods, we investigated the impact of varying environmental humidity conditions on the antibacterial and antiviral efficiency of materials. The temperature of the inoculation was set to 25°C, while the relative humidity was set to 15%, 50%, and 100%, respectively.

**Fig 5:**
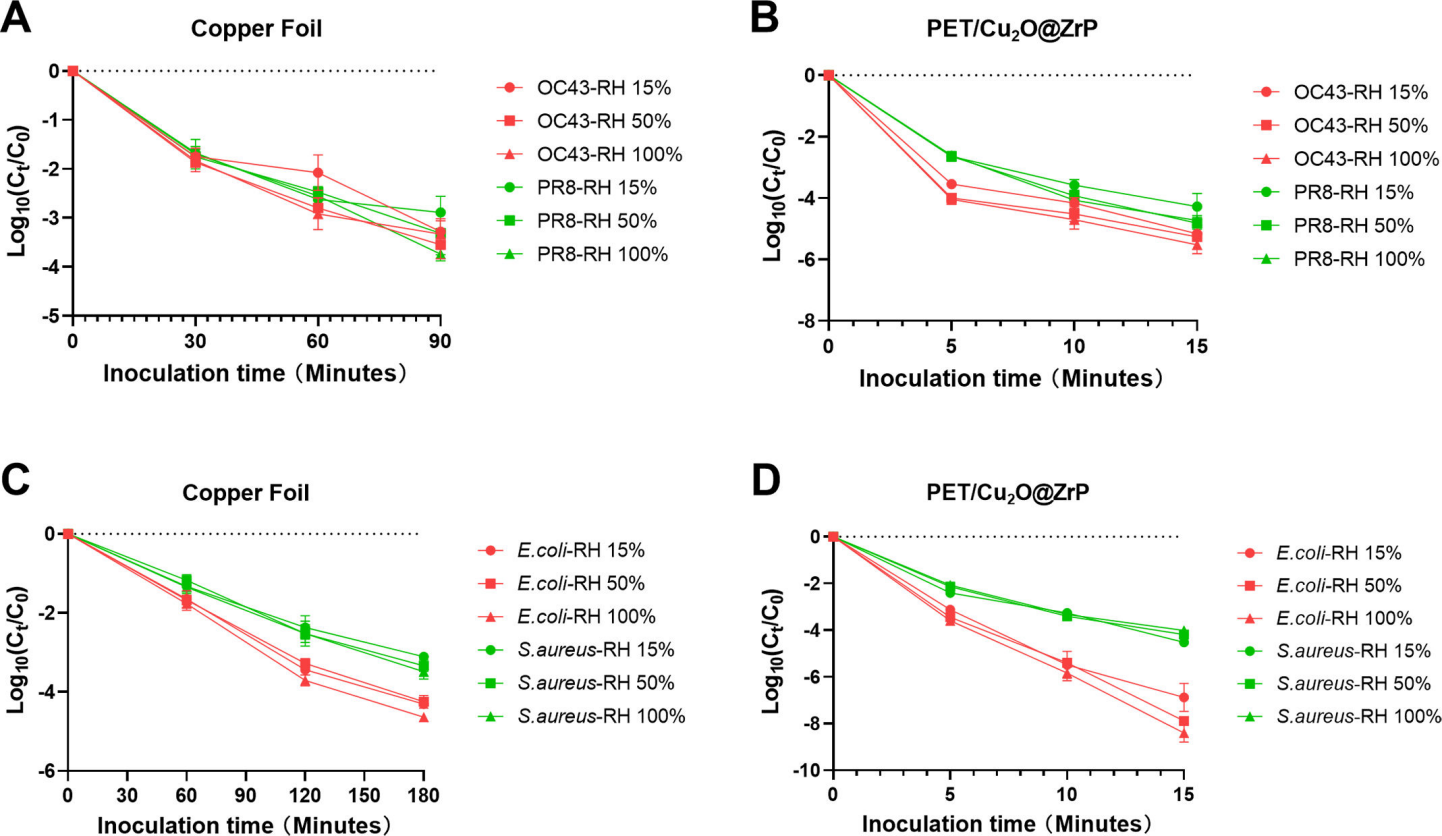
Environmental humidity has an influence on the antimicrobial testing results of material surfaces. (**A and B**) There is no significant difference in the antimicrobial testing results by different ambient humidity, either on non-porous or porous antimicrobial material surfaces. (**C and D**) Antimicrobial test results are not affected by humidity when using standard testing methods.

First, we modified the environmental humidity conditions during inoculation based on the ISO 21702 methodology to assess how humidity levels influence the virus-killing performance of copper foil. The results demonstrated that both viruses were significantly inhibited within 90 min on the copper foil surface, with OC43 showing a more pronounced decline compared to PR8. However, varying humidity conditions did not significantly alter the antiviral efficacy of the copper foil (Fig. 5A). Using the ISO 18184 methodology and setting the relative humidity conditions during inoculation to 15%, 50%, and 100%, we found that PET/Cu_2_O@ZrP exhibited faster antiviral effects under low humidity conditions compared to high humidity conditions. The virus-killing efficacy showed a significant difference across these humidity levels in 15 min (Fig. 5B).

To evaluate the influence of humidity on antibacterial results, we adjusted the environmental parameters in the ISO 22196 and ISO 20743 test methodologies by setting the ambient relative humidity to 15%, 50%, and 100%. Similar to the antiviral test results, changes in humidity did not significantly affect the antibacterial efficacy of copper foil across different humidity groups, although its efficacy varied against different bacterial strains (Fig. 5C). However, when the inoculation time was reduced to 15 min and the ambient relative humidity was adjusted to 15%, 50%, and 100%, the antimicrobial test results under the ISO 20743 methodology showed significant changes. Specifically, the material exhibited faster and more effective antibacterial activity against both *E. coli* and *S. aureus* under low humidity conditions (Fig. 5D).

The results from using ISO 21702 and ISO 18184 to evaluate the antiviral efficacy of materials, as well as ISO 22196 and ISO 20743 to assess their antibacterial efficacy, demonstrate that humidity has no significant impact on test results when non-porous structural materials with surface cover film are used. These findings differ from previous reports (33, 34), as those studies did not use a cover film on the inoculum, and the antimicrobial effects of copper in those cases did not rely on the release of ions into the inoculum but on a contact killing mechanism (35). However, in antimicrobial testing of porous materials, lower humidity levels were associated with stronger antibacterial activity but not antiviral activity. This difference may be attributed to the rapid antiviral effect of the PET/Cu_2_O@ZrP material, which occurs within 15 min. Over such a short duration, the impact of humidity on the inoculum is not significant enough to influence the results. In contrast, the antibacterial effect requires a longer duration of 90 min, during which environmental humidity can significantly affect the material’s antibacterial test results.

### Changes in bacterial ROS production levels after incubation with antimicrobial materials at different ambient temperatures and humidity

Many studies attribute the antibacterial activity of copper to its ability to rupture membranes, accumulate ions inside cells, inactivate proteins, and cause DNA damage (36). Additionally, copper is believed to induce oxidative stress by producing ROS), which is considered one of the major mechanisms of its antimicrobial efficacy. To verify whether the changes in the efficacy of copper-containing antimicrobial materials under different ambient temperature and humidity conditions are associated with ROS production, we tested the intracellular ROS levels in *E. coli* after inoculation on different material surfaces.

**Fig 6:**
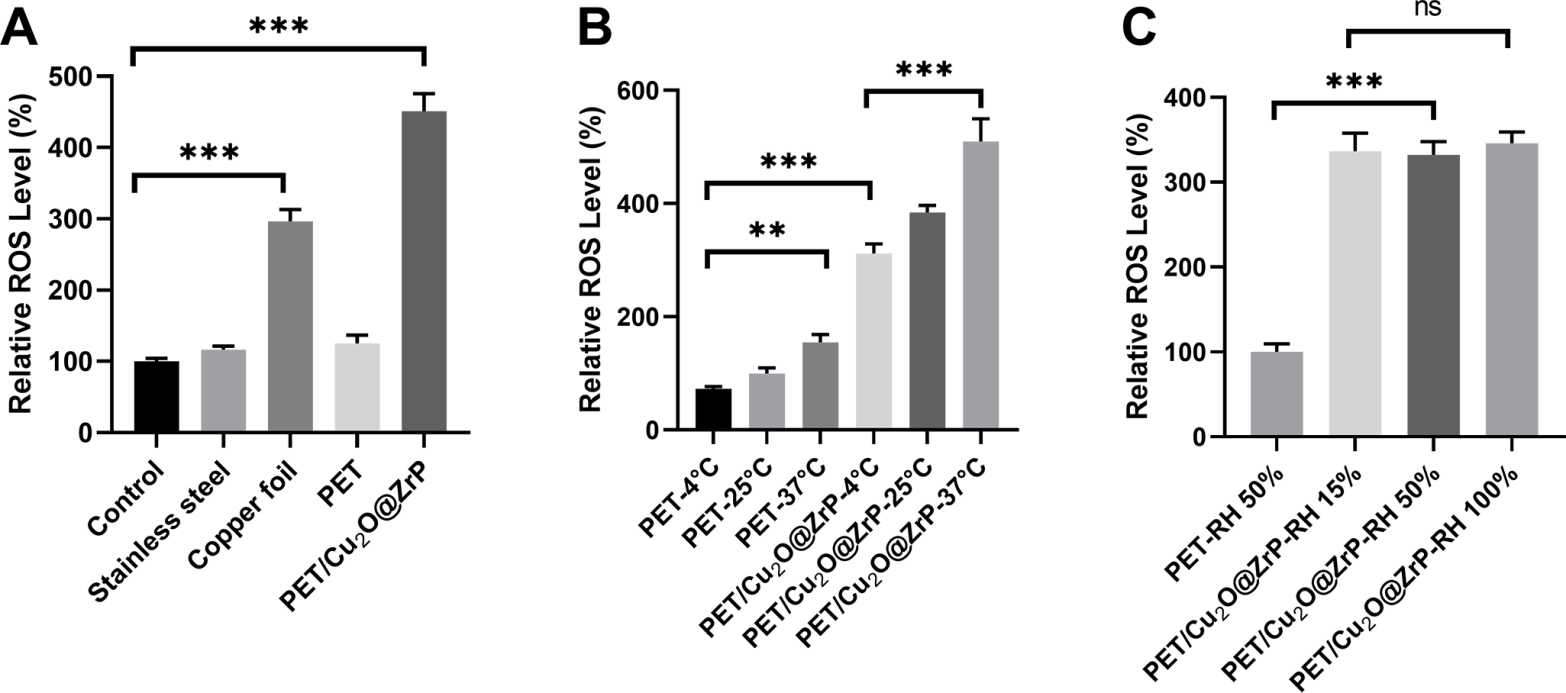
The antimicrobial efficacy results are consistent with the results of inducing ROS production. (**A**) Intracellular ROS production levels are higher on antimicrobial material surfaces. (**B**) Temperature can affect intracellular ROS levels on antimicrobial material surfaces. (**C**) Effect of ambient humidity on intracellular ROS levels on the surface of antibacterial materials. ns, non-significant, **P < 0.01, ***P < 0.001.

Firstly, we tested the ROS levels in *E. coli* cells after incubating them on the surfaces of four materials—stainless steel, copper foil, PET, and PET/Cu_2_O@ZrP—for 30 min at room temperature (25°C) and medium humidity (50% RH). After 30 min of incubation, intracellular ROS levels in *E. coli* on the copper foil surface increased significantly compared to those on the stainless steel surface. Similarly, intracellular ROS levels in *E. coli* on the PET/Cu_2_O@ZrP surface also increased significantly compared to those on the PET surface (Fig. 6A).

Next, we measured the intracellular ROS levels in *E. coli* after incubating them with PET/Cu_2_O@ZrP materials for 30 min under different temperature conditions. Although the intracellular ROS level of *E. coli* on the PET/Cu_2_O@ZrP surface incubated at a low temperature (4°C) was significantly higher than that of bacteria on the PET surface at 25°C, it remained lower than the ROS level in cells on PET/Cu_2_O@ZrP material at room temperature. In contrast, *E. coli* on PET/Cu_2_O@ZrP had the highest ROS levels when incubated at 37°C (Fig. 6B).

To investigate whether the differences in antimicrobial efficacy of materials caused by changes in ambient humidity are related to ROS production, we measured the intracellular ROS levels in *E. coli* cells on the surfaces of PET and PET/Cu_2_O@ZrP materials after incubation at various humidity levels for 30 min at 25°C. The results showed that environmental humidity significantly impacts intracellular ROS levels in *E. coli* cells inoculated with PET/Cu_2_O@ZrP (Fig. 6C). Specifically, lower environmental humidity led to higher ROS levels in the bacteria.

These findings suggest that the antibacterial effect of copper-containing materials may be related to increased intracellular ROS in microbes, consistent with previous research. Moreover, the effects of temperature and humidity on intracellular ROS further indicate that the varying levels of ROS produced by microbes in response to antimicrobial materials under different environmental conditions may be a significant factor influencing the antibacterial efficacy of these materials.

## DISCUSSION

Different technical standards institutions have published a series of standard methods for testing the antibacterial and antiviral properties of materials. For non-porous surfaces, antibacterial testing methods include ISO 22196, JIS Z 2801, and ISO 7581. For porous materials, such as fabric, thread, and sponges, ISO 20743, AATCC 100, and JIS L 1902 are frequently used (37). For antiviral tests, ISO 21702 is used for non-porous surfaces, while ISO 18184 is designed for porous textiles. Among these testing methodologies, ISO 22196, ISO 20743, ISO 21702, and ISO 18184 are widely recognized and used globally.

However, these testing methods have more or less shortcomings. For example, it has been noted that the ISO 22196 method is insufficient for testing the antibacterial performance of material surfaces under dry conditions. As a result, the ISO 7581 testing method, which incubated the inoculated specimens at a RH of 30%–65% was introduced in recent years to address this limitation. These insufficiencies suggest that previous antimicrobial testing methodologies do not account for the impact of temperature, humidity, light, and other environmental conditions on test results, potentially leading to misjudgments of the antimicrobial properties of materials. Consequently, antimicrobial materials that perform well in these standard tests often fail to demonstrate effectiveness in practical applications and clinical settings, failing to provide clinical benefits to users. This has led to many products not gaining approval from regulatory bodies, such as the FDA or NMPA. Therefore, it is necessary to improve existing antimicrobial standard testing methods by incorporating control factors, such as incubation time span, temperature, and humidity, into the current methodologies.

We believe that revising environmental parameters, such as humidity and temperature, in the existing ISO standard methodologies may significantly alter our judgment on the antibacterial performance of materials. Thus, understanding the impact of temperature or humidity on the testing results of antimicrobial efficacy based on each ISO methodology has significant practical importance. It helps us recognize the shortcomings of existing testing methodologies and guide the improvement of these methodologies by adapting the environmental conditions to the protocol. To the author’s knowledge of the research progress, past studies have not systematically explored how modifications to environmental parameters—based on traditional standard antimicrobial testing methods—affect the determination of antibacterial efficacy.

In commonly used antimicrobial testing methodologies, the incubation time is typically set at around 24 h. Although only ISO 18184 allows for a shorter incubation time of just 2 h, this still falls short of the rapid action required for medical antimicrobial materials. In clinical settings, particularly for personal protective equipment, surgical drapes, and other medical devices, the service time of these products is often only a few hours. If an antimicrobial material cannot exhibit antimicrobial activity within tens of minutes, it is reasonable to question its clinical relevance. Thus, we need instant killing antimicrobial materials and corresponding testing methods (38). This study demonstrates that effective antibacterial materials can achieve an antimicrobial efficiency of over 99% within tens of minutes, or even faster. These findings suggest that future revisions of standards should consider establishing classifications for antimicrobial efficiency based on the speed of antibacterial action—namely, rapid antimicrobial testing (within minutes), medium-speed antimicrobial testing (within hours), and slow antimicrobial testing (over tens of hours).

Existing standard testing methods for bacteria often use relatively high incubation temperatures, such as 35°C or 37°C, while antiviral tests typically use 25°C. However, the environmental temperatures at which antimicrobial materials are clinically used can vary widely, from cold outdoor environments in winter to high-temperature settings like food processing plants. To determine whether current antibacterial and antiviral testing standards can accurately present the properties of materials under changing ambient temperatures, we tested microbial survival on material surfaces and the efficacy of antimicrobial materials at different ambient temperatures ranging from 4°C to 37°C. Our study found that both material surfaces without antimicrobial activity and antimicrobial material allow bacteria and viruses to survive longer at lower ambient temperatures. In previous studies, it has been demonstrated that temperature significantly influences the survival of bacteria and viruses on material surfaces. However, only a few studies have attempted to discover the effect of temperature on the efficacy testing results of antimicrobial materials. In some studies, it was shown that citric acid could not sufficiently inactivate on material surface at −10°C (39). Other scholars conducted ISO 22196 tests at 20°C–25°C and the standard-required temperature of 35°C, concluding that higher antibacterial activity values were obtained at 35°C (40). Generally, higher temperatures correlate with shorter survival times for these microorganisms. There is consensus in the research regarding the effect of temperature on microbial survival. Numerous studies have concluded that, regardless of whether the surface is porous or non-porous and whether it contains antibacterial active ingredients, lower incubation temperatures result in longer microbial survival times and reduced antibacterial activity. This raises an important question: Does the use of a 37°C incubation temperature in antibacterial testing overestimate the material’s antibacterial efficacy? Similarly, does incubating viruses at 25°C and bacteria at 37°C fail to accurately reflect the true antiviral and antibacterial efficacy of materials, especially when they are used in real-world scenarios, such as outdoors in winter or in cold chain applications? Our result indicates that existing standard testing methods fail to accurately present the antibacterial and antiviral efficiency of materials under different temperature conditions, especially under low-temperature conditions. This result suggests that different test temperature conditions need to be incorporated into the new material antimicrobial standards so that the material’s antibacterial efficacy test results will be consistent with the actual application efficacy.

Environmental humidity also impacts the survival of germs on material surfaces, as well as test results. On the surfaces of porous or non-porous materials without antibacterial activity, the long-term (several days) survival rate of microorganisms is influenced by humidity levels, with lower humidity leading to longer survival rates. For instance, in the study by Qian et al., the impact of humidity on antiviral efficacy was assessed by incubating 10 µL virus-containing droplets (without a film cover) on the material surface. Multiple studies have also shown that high humidity generally has a protective effect on the survival of microorganisms on material surfaces (41). Conversely, some research suggests that microorganisms survive longest under moderate humidity conditions, with both higher and lower humidity levels being less conducive to their survival. In contrast, our approach involved using standard antibacterial testing methods with a film cover on non-porous material surfaces and directly incubating samples on porous materials without a film cover. We found that humidity has a more pronounced effect on the results of testing methods for porous materials without film covers, whereas for samples covered with films, ambient humidity had no significant impact on the test results for the materials, which have fast antimicrobial effects in minutes. However, our results suggest that on surfaces of materials without instant kill effects, humidity has a significant impact on antimicrobial test results, and for materials surfaces with copper, the lower environmental humidity is associated with the lower survival of microbes. This implies that existing ISO standard testing methods, which control humidity at 90% (such as ISO 22196 and ISO 21702), may underestimate the antimicrobial properties of materials. This suggests that in future antimicrobial testing standards, incorporating different humidity levels into environmental parameters can better present the antimicrobial activity of materials in actual use.

Furthermore, the possible mechanisms of how these environmental factors affect antimicrobial activity were studied by testing the effects of materials on ROS production during antimicrobial incubation. The trends of intracellular ROS production levels and efficacy data under different environmental conditions are consistent, suggesting that environmental factors can be related to their main mechanism of copper-containing materials.

To the best of the authors' knowledge, this study represents the first systematic evaluation of the effects of environmental factors, including temperature and humidity, on antimicrobial test results, rather than directly adopting the temperature and humidity settings specified in current ISO standard test methods. Importantly, our research demonstrates that among various commonly used ISO standard testing methods, both non-porous surfaces (represented by metallic copper surfaces) and porous surfaces (represented by cuprous oxide–modified fiber PET/Cu_2_O@ZrP) exhibit antimicrobial activities that are influenced by temperature. Regarding humidity, its impact on antimicrobial activity and efficiency during testing is negligible for non-porous surfaces covered with a film within tens of minutes. However, on porous material surfaces, a slightly longer incubation time (hours) results in a significant difference. This may be attributed to the increased evaporation rate of the inoculated microbial solution and enhanced contact with the copper surface, thereby boosting the contact-killing antimicrobial efficacy of the copper material. These findings suggest that the influence of ambient temperature and humidity on material efficiency should be fully considered in future antimicrobial testing methodologies.

Some limitations of this study include the following: the antimicrobial testing methods were confined to four commonly used ISO standard methods, which do not fully capture the diversity of existing testing methods under varying temperature and humidity conditions. The environmental temperature range examined in this study did not include sub-zero temperatures. In sub-zero environments, test sample droplets will freeze into ice, altering the likelihood of the material surface contacting and killing pathogenic microorganisms in the test samples. Additionally, there has been no further investigation into the effects of temperatures above 37°C on the antimicrobial function of materials, although higher temperatures are generally associated with faster microbial killing. Our study did not extend to other antibacterial/antiviral materials for verification. Thus, it only reflects the impact of humidity and temperature changes on the antimicrobial efficacy of copper-containing materials. Whether the antibacterial efficacy of other materials is similarly affected requires further verification. Another shortcoming pertains to the methodology used for testing antimicrobial efficacy on non-porous materials. We did not employ an improved method that involves multi-point inoculation of micro-droplets without a film cover. If this non-film covering method had been used, ambient humidity might have influenced the antimicrobial test results on non-porous material surfaces.

## Data Availability

The data sets generated and analyzed during the current study are available from the corresponding author on reasonable request.
